# Supplementary material for: Time-series transcriptome analysis identified differentially expressed genes in broiler chicken infected with mixed Eimeria species
Source: Front Genet. 2022 Aug 8;13:886781. doi: 10.3389/fgene.2022.886781 (PMC9393255; doi:10.3389/fgene.2022.886781)
Supplement: Supplementary file 2 [file DataSheet1.ZIP › 7dpi_GO.Gsea.1625106495923/GOBP_NADH_DEHYDROGENASE_COMPLEX_ASSEMBLY.html]

Details for gene set GOBP\_NADH\_DEHYDROGENASE\_COMPLEX\_ASSEMBLY[GSEA]

|  || Dataset | TMM\_7dpi\_gct\_format\_7dpi\_gct\_format.Class\_4dpi.cls #PC\_versus\_NC.Class\_4dpi.cls #PC\_versus\_NC\_repos |
| Phenotype | Class\_4dpi.cls#PC\_versus\_NC\_repos |
| Upregulated in class | 1 |
| GeneSet | GOBP\_NADH\_DEHYDROGENASE\_COMPLEX\_ASSEMBLY |
| Enrichment Score (ES) | 0.7304473 |
| Normalized Enrichment Score (NES) | 3.2290537 |
| Nominal p-value | 0.0 |
| FDR q-value | 0.0 |
| FWER p-Value | 0.0 |
Table: GSEA Results Summary

  

Fig 1: Enrichment plot: GOBP\_NADH\_DEHYDROGENASE\_COMPLEX\_ASSEMBLY      
 Profile of the Running ES Score & Positions of GeneSet Members on the Rank Ordered List

  

| SYMBOL | TITLE | RANK IN GENE LIST | RANK METRIC SCORE | RUNNING ES | CORE ENRICHMENT || 1 | NDUFAF3 | na | 148 | 1.148 | 0.0341 | Yes |
| 2 | NDUFA2 | na | 303 | 0.935 | 0.0590 | Yes |
| 3 | NDUFB6 | na | 307 | 0.933 | 0.0965 | Yes |
| 4 | NDUFS5 | na | 308 | 0.932 | 0.1342 | Yes |
| 5 | NDUFB7 | na | 323 | 0.915 | 0.1700 | Yes |
| 6 | NDUFB2 | na | 348 | 0.885 | 0.2038 | Yes |
| 7 | NDUFAB1 | na | 356 | 0.876 | 0.2386 | Yes |
| 8 | NDUFA7 | na | 371 | 0.857 | 0.2721 | Yes |
| 9 | NDUFC2 | na | 394 | 0.834 | 0.3040 | Yes |
| 10 | NDUFA11 | na | 497 | 0.746 | 0.3256 | Yes |
| 11 | NDUFA13 | na | 557 | 0.708 | 0.3493 | Yes |
| 12 | NDUFS3 | na | 623 | 0.675 | 0.3711 | Yes |
| 13 | NDUFB3 | na | 656 | 0.657 | 0.3950 | Yes |
| 14 | NDUFS8 | na | 661 | 0.653 | 0.4211 | Yes |
| 15 | NDUFS6 | na | 686 | 0.642 | 0.4450 | Yes |
| 16 | NDUFA1 | na | 742 | 0.616 | 0.4654 | Yes |
| 17 | NDUFA5 | na | 794 | 0.593 | 0.4851 | Yes |
| 18 | NDUFAF8 | na | 894 | 0.557 | 0.4993 | Yes |
| 19 | NDUFB5 | na | 902 | 0.554 | 0.5212 | Yes |
| 20 | NDUFV3 | na | 988 | 0.527 | 0.5354 | Yes |
| 21 | NDUFB4 | na | 1015 | 0.517 | 0.5541 | Yes |
| 22 | NDUFA12 | na | 1023 | 0.513 | 0.5742 | Yes |
| 23 | NDUFA6 | na | 1079 | 0.492 | 0.5895 | Yes |
| 24 | NDUFA8 | na | 1080 | 0.492 | 0.6094 | Yes |
| 25 | NDUFS2 | na | 1124 | 0.477 | 0.6251 | Yes |
| 26 | NUBPL | na | 1139 | 0.472 | 0.6431 | Yes |
| 27 | NDUFB10 | na | 1215 | 0.450 | 0.6550 | Yes |
| 28 | NDUFV1 | na | 1307 | 0.428 | 0.6647 | Yes |
| 29 | NDUFB8 | na | 1330 | 0.423 | 0.6799 | Yes |
| 30 | NDUFS7 | na | 1371 | 0.412 | 0.6933 | Yes |
| 31 | NDUFV2 | na | 1395 | 0.408 | 0.7078 | Yes |
| 32 | ACAD9 | na | 1483 | 0.385 | 0.7161 | Yes |
| 33 | ECSIT | na | 1497 | 0.382 | 0.7304 | Yes |
| 34 | NDUFB9 | na | 1842 | 0.299 | 0.7138 | No |
| 35 | NDUFB1 | na | 1995 | 0.272 | 0.7121 | No |
| 36 | BCS1L | na | 2225 | 0.232 | 0.7023 | No |
| 37 | TIMM21 | na | 2269 | 0.226 | 0.7079 | No |
| 38 | TAZ | na | 2298 | 0.223 | 0.7145 | No |
| 39 | NDUFAF6 | na | 2404 | 0.207 | 0.7141 | No |
| 40 | NDUFA9 | na | 2879 | 0.141 | 0.6802 | No |
| 41 | NDUFA10 | na | 3412 | 0.073 | 0.6386 | No |
| 42 | NDUFS1 | na | 3763 | 0.033 | 0.6107 | No |
| 43 | TMEM126A | na | 3795 | 0.029 | 0.6093 | No |
| 44 | TIMMDC1 | na | 3850 | 0.022 | 0.6057 | No |
| 45 | NDUFAF1 | na | 3921 | 0.014 | 0.6004 | No |
| 46 | NDUFS4 | na | 4402 | -0.040 | 0.5618 | No |
| 47 | NDUFAF5 | na | 5835 | -0.184 | 0.4495 | No |
| 48 | FOXRED1 | na | 6248 | -0.229 | 0.4244 | No |
| 49 | NDUFAF4 | na | 7678 | -0.381 | 0.3203 | No |
| 50 | NDUFAF2 | na | 8545 | -0.491 | 0.2677 | No |
| 51 | NDUFAF7 | na | 8945 | -0.541 | 0.2563 | No |
Table: GSEA details [plain text format]

  

Fig 2: GOBP\_NADH\_DEHYDROGENASE\_COMPLEX\_ASSEMBLY      
 Blue-Pink O' Gram in the Space of the Analyzed GeneSet

  

Fig 3: GOBP\_NADH\_DEHYDROGENASE\_COMPLEX\_ASSEMBLY: Random ES distribution      
 Gene set null distribution of ES for **GOBP\_NADH\_DEHYDROGENASE\_COMPLEX\_ASSEMBLY**
